# Jaundice-predominant manifestation of Kawasaki disease in children

**DOI:** 10.3389/fped.2023.1281909

**Published:** 2024-01-09

**Authors:** Ya-Ning Huang, Chien-Yu Lin, Hsin Chi, Nan-Chang Chiu, Daniel Tsung-Ning Huang, Lung Chang, Yen-Hsin Kung, Ching-Ying Huang

**Affiliations:** ^1^ Department of Pediatrics, Hsinchu Municipal MacKay Children’s Hospital, Hsinchu, Taiwan; ^2^ Department of Pediatrics, Hsinchu MacKay Memorial Hospital, Hsinchu, Taiwan; ^3^ Department of Medicine, MacKay Medicine College, Taipei, Taiwan; ^4^ Department of Pediatric Infectious Diseases, MacKay Children’s Hospital, Taipei, Taiwan; ^5^ Department of Pediatrics, Tamshui MacKay Memorial Hospital, Taipei, Taiwan

**Keywords:** jaundice, IVIG-refractory disease, Kawasaki disease, children, hyperbilirubinemia

## Abstract

**Background:**

A jaundice-predominant presentation of Kawasaki disease (KD) is atypical.

**Methods:**

A total of 12 children with KD with a predominant manifestation of jaundice at MacKay Children's Hospital were reviewed, along with 42 cases reported in the literature since 1990.

**Results:**

The median age of the 12 patients was 1.85 years (range: 3 months–4 years), and 66.6% were male. All of the patients had elevated liver function at presentation, 50% had hydrops of the gallbladder, and almost 60% had gastrointestinal symptoms and signs. Complete KD was evident in 11 of the 12 patients (91.7%), and two patients (16.7%) had recurrent episodes. All of the patients received intravenous immunoglobulin (IVIG); however, one-third were refractory to treatment. Corticosteroids were used in five (41.7%) of the patients. Three (25%) of the patients had shock, and seven (58.3%) had coronary artery abnormalities, of whom one (8.3%) had persistent coronary artery aneurysm and the others recovered. A review of the 42 cases in the literature showed that the children with a jaundice-predominant presentation of KD had high rates of IVIG-refractory disease (25%), coronary artery abnormalities (25%), shock (13.2%), and corticosteroid treatment (24.2%).

**Conclusions:**

Children with KD presenting with a jaundice-predominant manifestation are at a higher risk of IVIG-refractory disease, coronary artery abnormalities, and more recurrent episodes. Physicians should be aware of the risk of shock in this population.

## Introduction

Up to 48.4% of patients with Kawasaki disease (KD) have an incomplete manifestation, and an atypical presentation may delay an accurate diagnosis (1, 2). Among the atypical presentations, hepatobiliary abnormalities during the clinical course of KD are rare. Burns et al. reported that hydrops of the gallbladder and hepatobiliary malfunction are closely related to the acute stage of KD (3), and Falcini et al. and Grewal et al. both reported acute febrile jaundice as an initial presentation of KD (4, 5). An atypical presentation of jaundice often occurs in the early stage of KD; however, the typical presentation can manifest 14 days after the first manifestations of jaundice (5). Some studies have reported an association between jaundice-predominant manifestations of KD during the acute stage with an increased risk of developing coronary artery abnormalities. Therefore, an early diagnosis of acute febrile jaundice in patients with KD could allow for prompt treatment to reduce the risk of fatal heart complications.

The aim of this study was to report the clinical manifestations, laboratory findings, imaging study findings, management, outcomes, and a literature review of children with KD presenting with jaundice-predominant manifestations.

## Materials and methods

In this retrospective case study, we performed a chart review of all patients with jaundice confirmed by laboratory findings (serum total bilirubin > 2 mg/dl) and diagnosed with KD between 2013 and 2021 at MacKay Children's Hospital. This level of serum total bilirubin was chosen based on the 21st edition of the Nelson Textbook of Pediatrics for clinically apparent jaundice in infants, children, and adolescents, which states a level of 2–3 mg/dl (34–51 µmol/L). Complete KD was defined as fever plus the presence of ≥4 principal clinical features: non-exudative conjunctivitis, oral lesions, neck lymphadenopathy, changes in the extremities, and skin rash. Those who had fever plus <4 principal clinical features with compatible laboratory or echocardiographic findings were defined as having incomplete (atypical) KD according to the 2017 American Heart Association definition (6).

Data on the clinical presentation and course were collected from electronic medical records, including sex, age, days of illness at admission, clinical characteristics at admission, initial imaging studies, laboratory tests, echocardiographic data, treatment, and outcomes. Aspirin treatment was recorded in actual dose per kg. The timing of steroid therapy for patients with KD was classified as primary steroid treatment including intravenous immunoglobulin (IVIG) and rescue steroid treatment for IVIG-resistant KD (7–9). Outcomes were classified as IVIG-refractory disease, recurrent KD (>2 weeks from the first episode), and coronary artery abnormalities determined at the following times: before IVIG (days from onset < 10 days), maximum *Z* score after IVIG (days from onset), and long-term follow-up (>4 months). Clinical characteristics were recorded, including complete or incomplete KD, jaundice (icteric skin), shock, vomiting, diarrhea, and hepatomegaly. The initial imaging study was conducted using abdominal ultrasound. Cutoff values for abnormal blood parameters were defined according to the patient's age, as follows: anemia (hemoglobulin < 5.83 mmol/L for 2- to 3-month-old infants; <6.89 mmol/L for 6-month-old infants; < 6.52 mmol/L for infants aged 6 months to 2 years; and <7.14 mmol/L for children aged 2–6 years), leukocytosis (white blood cell count > 14 × 10^9^/L), thrombocytopenia (platelet count < 140 × 10^9^/L), and elevated liver function (aspartate aminotransferase, AST > 35 U/L; alanine aminotransferase, ALT > 45 U/L).

A literature review was performed based on PubMed, Medline, and Embase, using the search terms: “Kawasaki AND jaundice AND children,” “Kawasaki AND hyperbilirubinemia AND children,” “Kawasaki AND jaundice AND infant,” “Kawasaki AND hyperbilirubinemia AND infant”, “Kawasaki AND liver failure AND children,” “Kawasaki AND hepatic encephalopathy AND children,” “Kawasaki AND hepatic dysfunction AND children,” from 1990 to December 2021. Only English-language articles were included. Cases of KD in the literature review were identified according to the clinical presentation and echocardiography, and a serum bilirubin level >2 mg/dl for jaundice. The relevant articles were reviewed, and the data on age, sex, clinical presentation, physical examinations, imaging studies, laboratory findings, management, and outcomes were extracted. The descriptive statistics are presented in tables with number, mean, median, percentage, and range. This study was approved by the MacKay Memorial Hospital Institutional Review Board (18MMHIS183e).

## Results

During the study period, 12 patients with jaundice and KD were identified in the general ward, newborn center, and pediatric intensive care unit. All patients presented with icteric skin and a serum bilirubin level >2 mg/dl. There were eight male patients (66.6%) and four female patients, with a median age of 1.85 years (range: 3 months to 4 years) (Table 1). In addition, the incidence rate was 2.7% (95% confidence interval: 0.013–0.049) at our hospital during 1 January 2013, to 31 December 2019.

**Table 1:** Clinical characteristics and outcomes of the 12 children with Kawasaki disease and jaundice.

	Patient No.					
	1	2	3	4	5	6
Demographic profile						
Sex	Female	Male	Male	Male	Female	Female
Age (years + months)	1 + 10	0 + 9	0 + 3	1 + 5	1 + 6	2 + 3
Days of illness at admission	1	4	2	2	3	4
Treatment and outcome						
Aspirin (mg/kg/day)	55	85	60	80	45	51
Steroids	−	−	−	Dexamethasone (rescue)	−	Methylprednisolone (rescue)
Days of illness until IVIG	3	5	3	3	6	4
IVIG refractory disease	No	No	No	No	No	Yes
Coronary artery abnormality	Yes	No	Yes	No	No	Yes
Before IVIG (days from onset, <10 days)^a^	Right coronary artery (*Z* + 2.31) (3)	Normal	Left main coronary artery (*Z* + 2.84) (3)	Normal	Normal	Normal
Maximum *Z* score after IVIG (days from onset)	Normal	Normal	Normal	Normal	Normal	Right coronary artery (*Z* + 4.08) (16)
Long-term follow-up (>4 months)	Normal	Normal	Normal	Normal	Normal	Normal
Recurrence (>2 weeks)	−	−	−	−	−	−
Complication	No	No	No	No	No	No
Outcome	Complete recovery	Complete recovery	Complete recovery	Complete recovery	Complete recovery	Complete recovery
Clinical characteristics at admission						
Completed KD	+	+	+	+	+	+
Fever >5 days	+	+	+	+	+	+
Non-exudative conjunctivitis	+	+	+	+	+	+
Rash	+	+	+	+	+	+
Extremity change	+	+	+	+	+	+
Oral lesion	+	+	+	+	+	+
Neck lymphadenopathy	−	−	−	+	−	+
Jaundice (icteric skin)	+	+	+	+	+	+
Shock	−	−	−	−	−	−
Vomiting/diarrhea	−/−	−/−	−/−	−/+	−/−	+/+
Hepatomegaly	−	−	−	−	+	−
Initial imaging study and laboratory tests						
Abdominal ultrasound (hydrops gallbladder)	−	+	−	−	+	+
CRP (mg/L)/ESR (mm h)	49/92	NA/38	165/NA	30.2/NA	126/NA	106/81
AST/ALT/gamma-glutamyl transferase (IU/L)	224/315/129	239/189/NA	520/419/NA	744/840/116	451/129/NA	59/61/NA
Blood urea nitrogen(mmol/L)/creatinine (µmol/L)	NA/NA	2.86/35.4	NA/NA	4.64/35.4	2.61/45.1	NA/NA
Bilirubin (total/direct) (µmol/L)	58.2/27.4	54.7/NA	61.6/41	42.8/27.4	23.9/12	71.8/51.3
Highest bilirubin (total/direct) (µmol/L)	47.9/35.9					
White blood cell count (10^9^/L)	11.8	11.9	7.5	12.8	17	9.3
Hemoglobin (mmol/L)	7.45	6.64	6.14	6.02	6.21	6.76
Platelet count (10^9^/L)	258	368	122	275	314	208
Sodium (mmol/L)/albumin (g/L)	NA/43	136/43	NA/35	139/40	134/34	128/37

	Patient No.					
	7	8	9	10	11	12
Demographic profile						
Sex	Male	Male	Female	Male	Male	Male
Age (years + months)	1 + 7	2 + 2	2 + 3	4	0 + 11	3 + 4
Days of illness at admission	5	3	3	5	3	4
Treatment and outcome						
Aspirin (mg/kg/day)	30	40	50	70	2	3
Steroids	−	−	Methylprednisolone (rescue)	Methylprednisolone (primary combination)	−	Methylprednisolone (rescue)
Days of illness until IVIG	5	5	7	5	9	5
IVIG refractory disease	No	Yes	No	Yes	No	Yes
Coronary artery abnormality	No	No	Yes	Yes	Yes	Yes
Before IVIG (days from onset, <10 days)^a^	Normal	Normal	Pericardial effusion (4,7)	Normal	Right coronary artery (*Z* + 2.0) (8)	Left main coronary artery (Z + 2.66) (5)
Maximum *Z* score after IVIG (days from onset)	Normal	Normal	Right coronary artery (*Z* + 3.45)	Right coronary artery (*Z* + 2.98) (14)	Normal	Left main coronary artery (*Z* + 4.13) (14)
			Left main coronary artery (Z +2.91) (17)	Left main coronary artery (Z + 3.66) (20)		
Long-term follow-up (>4 months)	Normal	Normal	Right coronary artery (*Z* + 3.14) (2 years)	Normal (3 years)	Normal	Normal (4.5 months)
Recurrence (>2 weeks)	−	−	+	−	−	+
Complication	No	No	Acute hepatitis, suspect aspirin related	No	No	Acute kidney injury
Outcome	Complete recovery	Complete recovery	Persistent coronary aneurysm	Complete recovery	Complete recovery	Complete recovery
Clinical characteristics at admission						
Completed KD	+	+	+	+	−	+
Fever >5 days	+	+	+	+	+	+
Non-exudative conjunctivitis	+	+	−	+	−	+
Rash	+	+	+	+	+	+
Extremity change	+	+	+	+	+	+
Oral lesion	+	+	+	+	+	+
Neck lymphadenopathy	−	+	+	+	−	+
Jaundice (icteric skin)	+	+	+	+	+	+
Shock	−	−	+	−	+	+
Vomiting/diarrhea	−/−	+/−	+/+	+/+	−/−	+/−
Hepatomegaly	−	−	−	−	−	+
Initial imaging study and laboratory tests						
Abdominal ultrasound (hydrops gallbladder)	NA	NA	+	+	+	−
CRP (mg/L)/ESR (mm h)	34/43	64/69	192/96	151/48	141/13	437/88
AST/ALT/GGT (IU/L)	51/9/NA	103/209/NA	355/323//156	64/187/NA	33/38/53	32/42/50
Blood urea nitrogen (mmol/L)/creatinine (µmol/L)	NA/NA	NA/NA	3.64/36.2	NA/NA	6.43/61.9	32.32/349.3
Bilirubin (total/direct) (µmol/L)	37.6/13.7	44.5/20.5	143.7/97.5	97.5/41	99.2/66.7	153.9/106
Highest bilirubin (total/direct) (µmol/L)	224.1/8.4					
White blood cell count (10^9^/L)	15.2	5.9	10	15.8	13.4	27.7
Hemoglobin (mmol/L)	6.89	6.89	6.14	8.01	6.89	6.76
Platelet count (10^9^/L)	376	405	299	268	284	247
Sodium (mmol/L)/albumin (g/L)	136/38	132/36	135/28	136/33	136/25	128/34

+ = present; − = absent, AST, aspartate aminotransferase; ALT, alanine aminotransferase; CRP, C-reactive protein; ESR, erythrocyte sedimentation rate; IVIG, intravenous immunoglobulin; NA, non-available.

^a^ Before IVIG defined as ±3 days.

### Clinical manifestations

The median duration of illness at admission was 3.3 days (range: 1–5 days). Complete KD was evident in 11 of the 12 patients (91.7%). Six patients (50%) presented without neck lymphadenopathy, and two patients (16.7%) did not have non-exudative conjunctivitis. Seven (58.3%) patients had gastrointestinal presentations of KD, including abdominal pain, vomiting, and diarrhea. Three (25%) patients had shock, and two (16.7%) patients had hepatomegaly. No bacteria grew in the blood or urine cultures, and serology tests excluded hepatitis A, B, and C viruses, adenovirus, herpes simplex virus, Epstein–Barr virus, and cytomegalovirus.

### Laboratory investigations and imaging studies

All 12 patients had elevated liver function and elevated C-reactive protein (CRP) at presentation. Hydrops of the gallbladder was found in six of the 12 (50%) patients, the median CRP level was 13.6 mg/dl, and the median serum AST/ALT levels were 239.6/230.0 U/L. Anemia (6/12, 50%), leukocytosis (4/12, 33.3%), thrombocytopenia (1/12, 8.33%), hyponatremia (3/10, 30%), and hypoalbuminemia (2/12, 16.7%) were also found in the patients. One case had KD shock syndrome complicated with acute kidney injury (creatine level: 3.95 mg/dl). The median total and direct bilirubin levels were 4.85 (range: 2.2–13.12) mg/dl and 3.06 (range: 0.8–8.44) mg/dl, respectively.

### Management and outcomes

All patients received aspirin treatment at a dosage between 2 and 85 mg/kg/day. In addition, all patients received IVIG at a median of 5 days from the onset of illness (range: 3–6 days). Five of the 12 patients (41.7%) received corticosteroid treatment, one received primary combination therapy with IVIG, and the others received corticosteroid rescue therapy owing to IVIG resistance. With regards to the outcomes, the median time to recover from jaundice was 7.09 days (range: 3–16 days), jaundice gradually attenuated with the improvement in KD, and all of the patients completely recovered from jaundice. Four (33.3%) patients had IVIG-refractory disease, and coronary artery abnormalities were detected in seven patients (58.3%) during follow-up. We monitored coronary artery lesions at the following time points: before IVIG (<10 days from onset), 2–3 weeks from fever onset, and >4 months in long-term follow-up. Coronary artery involvement was present in seven patients (58.3%), and maximum *Z* scores ranged from 2.98 to 4.13 between days 14 and 20 from the onset of fever. Only one patient had persistent coronary aneurysm, and the others had recovery after 4 months of long-term follow-up. Two patients (16.7%) had recurrent episodes of KD. The first had a prolonged recovery from jaundice of 20 days during the second KD episode, whereas jaundice improved rapidly within 3 days in the other patient. Complications occurred in two patients; the first had aspirin-related acute hepatitis with persistent coronary aneurysm; and the second had KD shock syndrome complicated with acute kidney injury after continuous veno-venous hemofiltration during the acute stage. All but one of the patients completely recovered from KD without further complications. The patient who did not completely recover had persistent coronary aneurysm (right coronary artery *Z* score: 3.14; left coronary artery *Z* score: 2.62) in the following 2 years.

### Literature review

**Figure 1:**
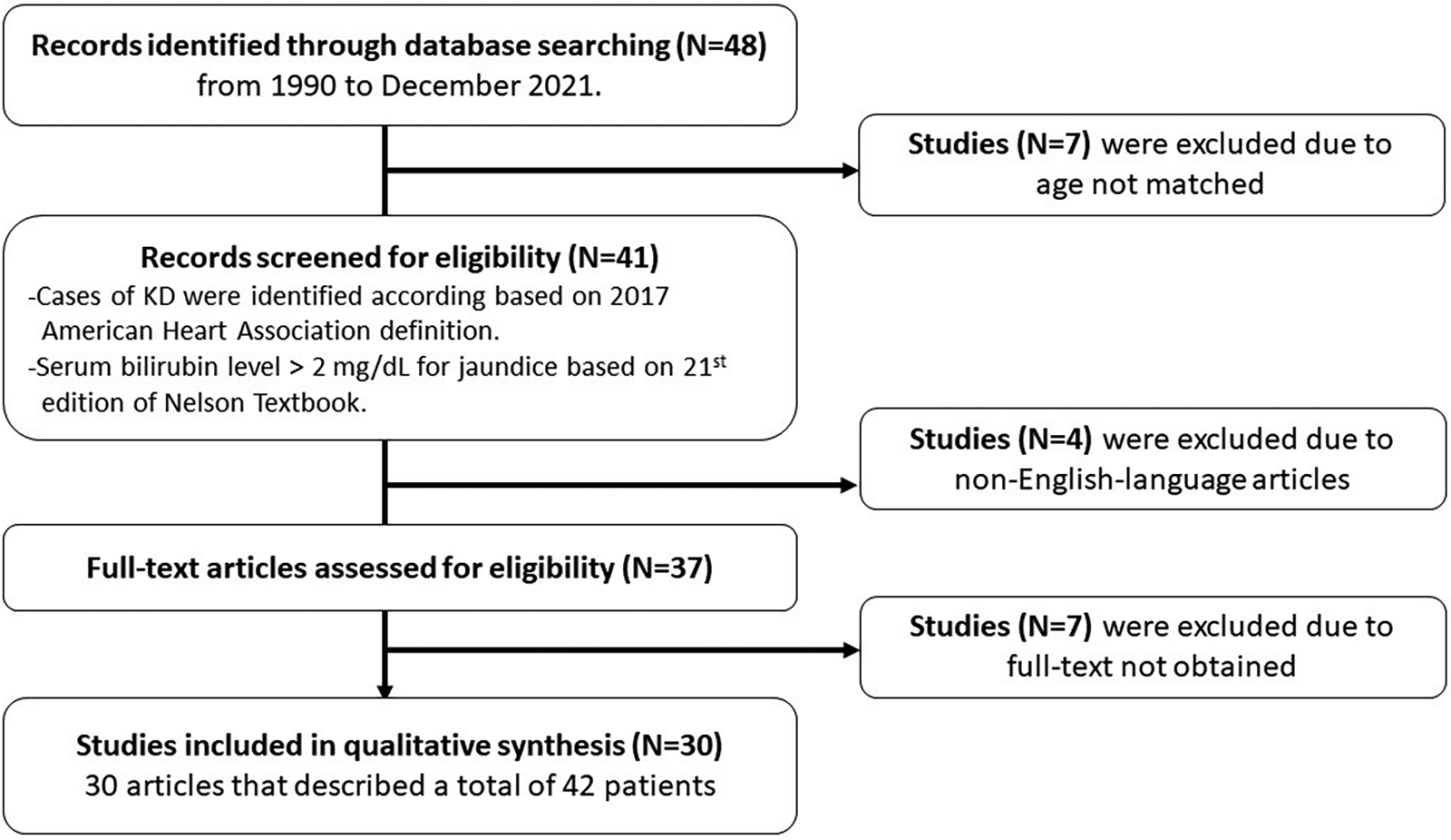
Flow diagram.

In the literature review, we identified 30 articles that described a total of 42 patients (Figure 1) who met the definition of the jaundice predominant manifestation of KD (Table 2). Their median age at onset was 7.0 years (range: 1–18 years), and 73% were male. On admission, 68.3% of the patients had complete KD, 50% of the patients had gastrointestinal symptoms, 64.9% had hepatomegaly, 73.7% had leukocytosis, 56.5% had hydrops of the gallbladder, and 97.2% had elevated liver function. In addition, 13.2% of the patients had shock. Regarding outcomes, 25% of the patients had IVIG-refractory disease, and 25% had coronary artery abnormalities. Regarding treatment, almost all of the patients received aspirin (97.5%), and more than one-fifth received steroid treatment (24.2%). Recurrence was not observed in any of the patients; however, 29.2% of the patients had complications. Ten patients had long-term coronary artery aneurysms, including one who received balloon angioplasty, one who had a medium right coronary artery aneurysm, and one who had a severe coronary lesion. This 12-year-old boy had two left coronary artery aneurysms 7 and 15 mm in length, a right coronary artery aneurysm 9 mm in length, and received hemofiltration therapy for acute kidney injury (creatinine level: 6.6 mg/dl). In addition, one 7-year-old boy died due to congestive heart failure, *Klebsiella pneumoniae* sepsis, and disseminated intravascular coagulopathy with a necrotic gallbladder (1).

**Table 2:** Comparison of the patients with Kawasaki disease and jaundice at MacKay Children's Hospital to those in the literature review.

	Patients from MCH (2013–2021) (*n* = 12)		Literature review (1990–2021) (*n* = 42)	
Demographic data				
Sex (male), *n/N* (%)	8/12	66.7	27/37	73.0
Age (years), median(range)	1.85	0.25–4	7.0	1–18
Outcome, *n*/*N* (%)				
IVIG-refractory disease	4/12	33.3	10/40	25
Coronary artery abnormality	7/12	58.3	10/40	25
Recurrence	2/12	16.7	0/10	0
Complication	2/12	16.7	12/41	29.2
Presentation, *n/N* (%)				
Completed KD	11/12	91.7	28/41	68.3
Shock	3/12	25	5/38	13.2
Gastrointestinal symptoms and/or signs	7/12	58.3	20/40	50
Hepatomegaly	2/12	16.7	24/37	64.9
Hydrops gallbladder	6/12	50	13/23	56.5
Leukocytosis	4/12	33.3	28/38	73.7
Thrombocytopenia	1/12	8.3	2/20	10.0
Elevated liver function	12/12	100	35/36	97.2
Management, *n/N* (%)				
Aspirin	12/12	100	39/40	97.5
Steroids	5/12	41.7	8/33	24.2

MCH, Mackay Children's Hospital; *n*, number; *N*, number; IVIG, intravenous immunoglobulin.

Complications: acute hepatitis, suspect aspirin related, acute kidney injury, persistent coronary artery aneurysms, hepatic encephalopathy, congestive heart failure, *Klebsiella pneumoniae* sepsis, and disseminated intravascular coagulopathy with a necrotic gallbladder and death.

## Discussion

Reports of atypical findings of KD have increased in the past few decades, and gastrointestinal symptoms are not easily recognized in the early stages of the disease. Although gastrointestinal symptoms are not included in the classical diagnostic criteria, hydrops of the gallbladder and acute febrile jaundice have been reported as atypical findings in some studies (10–12). Suhaini et al. suggested that it is crucial to focus on hepatobiliary manifestations in KD (13), because KD is the second most common cause of acute febrile jaundice and has been reported in up to 20% of cases (2, 14, 15). Our findings highlight the importance of the jaundice-predominant manifestation of KD in infants and children. An atypical presentation of jaundice often occurs in the early stage of KD, at an average of 3.3 days between the second and sixth day of the febrile course (15). However, the typical presentation can manifest as late as 14 days after the onset of the jaundice manifestation (5). Thus, the early recognition of febrile jaundice as the initial presentation of KD would allow for a more timely diagnosis.

The pathophysiology of jaundice among patients with KD is still unclear (16). The proposed mechanisms include vasculitis of hepatobiliary vessels, edematous change of the cystic duct wall, inflammation of the serosa of the liver and gallbladder, and cholangitis, which has been suggested to be the cause of cholestasis and hepatitis (17). Many studies have proposed that liver function tests can detect inflammation and predict the possibility of developing IVIG-refractory disease or coronary artery aneurysms (18–20).

Hydrops of the gallbladder has been reported in 5%–20% of KD patients, especially in the first 2 weeks (5, 10, 14). Although hydrops of the gallbladder has been reported to be a transient process in KD patients with jaundice (21), invasive surgery is still performed in some cases with severe hydrops to prevent further rupture (5). In addition, a severe presentation of atypical KD has been misdiagnosed as acute abdomen in some cases; therefore, if an accurate diagnosis of KD can be made earlier when the initial presentation is febrile jaundice, invasive interventions may be avoided.

KD is the leading cause of coronary artery disease in children, and a previous study reported the presence of coronary artery abnormalities from 9 to 18 days after the onset of fever (10). Another study reported that the incidence of coronary artery abnormalities decreased from 20%–25% to 2%–4% in patients who used IVIG in the initial 10 days (15). In addition, an atypical presentation of KD has been associated with a higher risk of coronary artery abnormalities (22). In KD, a higher rate of coronary artery abnormalities but a lower rate of prompt and complete diagnosis is a problem, especially among younger patients (23), as shown in our study. Moreover, up to 58% of our patients had a high CRP level, with a median CRP level of 136 mg/L. In addition, Newburger et al. reported that almost 18% of their KD patients with fever, high CRP level (>100 mg/L), jaundice, and elevated liver function had a poor response to IVIG (24). A proposed mechanism by which jaundice leads to IVIG-refractory disease or coronary abnormalities is a high level of inflammation, and this has been suggested to be the cause of cholestasis and hepatitis (17). Many studies have also proposed that the level of CRP can be used to detect inflammation and predict the possibility of developing KD. In addition, more severe inflammatory processes, as reflected by high CRP or hyperbilirubinemia, have been associated with a higher risk of developing IVIG-refractory disease or coronary artery aneurysms (25–27). Hence, a prompt and accurate diagnosis of KD with adequate treatment in the initial 10 days may alleviate the inflammatory conditions of KD such as gastrointestinal manifestations and reduce the risk of coronary artery aneurysms.

In addition to IVIG therapy, the optimal timing to initiate corticosteroid treatment is also critical. In our study, up to 40% of the patients received steroid treatment, and they all recovered from jaundice. Wardle et al. reported that initiating steroid treatment in the acute stage could decrease markers of inflammation, prevent coronary artery abnormalities, shorten the length of hospital stay, and improve clinical symptoms. Although evidence on the benefits of a long course of steroids is insufficient, many studies still support that steroid treatment should be given to high-risk patients with KD (9). In addition, several studies have suggested that corticosteroids may be beneficial for two groups of high-risk patients, namely, those with IVIG-resistant disease, and those with shock (7, 9). For IVIG-resistant patients, the recommended dose of corticosteroids as rescue therapy is 30 mg/kg/day for three consecutive days followed by oral tapering (28). For KD patients with shock, steroids are used as adjunctive therapy combined with IVIG as the primary treatment to reduce inflammation (29). Many studies have suggested an association between hepatobiliary dysfunction with a poor response to IVIG treatment and the presence of coronary aneurysms (30–33). Thus, to prevent the significant complication of coronary artery abnormalities, clinical guidelines from Japan and the United Kingdom suggest administering steroid treatment concomitantly with IVIG rather than IVIG alone (34).

The findings of the literature review and our cases suggest that jaundice may be an early finding of KD, especially among young infants. In addition, the younger children who presented with more atypical manifestations had a higher incidence of coronary artery abnormalities (23). Early IVIG treatment has been reported to reduce the occurrence of coronary artery abnormalities to below 5% (15). In our series, all of the patients received IVIG within 10 days, and all but one, with a persistent coronary aneurysm, completely recovered. Similarly, Keeling et al. concluded that acute febrile cholestasis without coronary artery lesions may delay the diagnosis and treatment and increase the potentially fatal risk of heart disease (35). Conversely, Taddio et al. found that some KD patients had jaundice but no hydrops of the gallbladder at presentation (14), whereas Sun et al. found that some KD patients with obstructive jaundice who progressed to severe hydrops of the gallbladder had a similar presentation with acute abdomen (36). It is therefore crucial to detect jaundice clinically (13). Taken together, our findings indicate that the timely suspicion of KD through the detection of jaundice can prevent further coronary artery complications and acute abdomen among young infants.

In this study, the patients with febrile jaundice were younger, with an average age of 1.85 years, and had a higher risk of IVIG-refractory disease and coronary artery abnormalities. By contrast, Fradin et al. (37) found that older male children and adolescents had a higher risk of presenting with incomplete KD and further complications. Furthermore, Vergine et al. (38) and Momenah et al. reported that older children above the age of 9 years accounted for 7.5% of their KD patients, and that they had a higher risk of coronary artery abnormalities (39), especially those who presented with febrile cholestasis. In addition, Pratap et al. found that hepatic-predominant manifestations were more common in older children who were more refractory to treatment (40). Therefore, some experts have concluded that a high level of suspicion should be maintained in children who present with febrile jaundice and atypical KD, especially among older children and adolescents. However, the mechanism has yet to be elucidated.

Some diseases such as scarlet fever, toxic shock syndrome, and Gilbert's syndrome with infectious signs may mimic KD, and their differential diagnoses should be excluded carefully (35). In addition, other possible causes of acute febrile jaundice including infections, metabolic and malignant diseases, and medications should also be excluded.

It is worth mentioning that aspirin should be used cautiously when managing KD patients with jaundice or hepatitis. Falcini et al. proposed that aspirin should not always be given to patients with jaundice for anti-inflammation because aspirin also has a significant choleretic effect (41). KD is highly associated with inflammation. All of our patients received aspirin treatment, and seven of the 12 (58%) patients received a dosage above 50 mg/kg/day. One of our patients (Patient No. 9 in Table 1) had complications of aspirin-related acute hepatitis and a coronary aneurysm, and the liver function and jaundice dramatically improved after we stopped aspirin. The patient completely recovered from hepatitis and jaundice after 16 days, and we gave dipyridamole for the persistent coronary aneurysm. Our study supports that aspirin should be used with caution, especially in children with jaundice or hepatitis, and that they should be followed and monitored closely during aspirin treatment.

The major limitation of this study is that some information was missing in the electronic medical records, including imaging studies, laboratory tests, follow-up treatment, and long-term outcomes. Consequently, only 12 patients who had complete records were enrolled. This was also seen in the literature review, and so only 39 patients were considered.

In summary, incomplete KD should be considered in children with febrile jaundice of unknown etiology, especially among young infants, despite the absence of coronary artery lesions. Given its safety and acceptability, IVIG therapy should be given early to prevent the development of heart problems. The rapid amelioration of the clinical condition after IVIG or steroid treatment confirms the suspicion of KD, especially the atypical type. Moreover, patients with jaundice may have a higher risk of IVIG-refractory disease and recurrence of KD.

## Conclusions

Our study supports that KD should be considered early in children who present with acute febrile jaundice as an initial manifestation, even without coronary artery abnormalities. Given its safety and acceptability, IVIG therapy should be given early. Furthermore, young infants and children with jaundice manifestations of KD have a higher risk of IVIG-refractory disease and coronary artery abnormalities. This manifestation appears to be more common in patients with KD shock syndrome and recurrent episodes of KD, especially when the patients are younger.

## Data Availability

The raw data supporting the conclusions of this article will be made available by the authors, without undue reservation.
